# Supplementary material for: Glucose and NAADP trigger elementary intracellular β-cell Ca2+ signals
Source: Sci Rep. 2021 May 21;11:10714. doi: 10.1038/s41598-021-88906-0 (PMC8140081; doi:10.1038/s41598-021-88906-0)
Supplement: Supplementary file 3 — Supplementary Information. [file 41598_2021_88906_MOESM3_ESM.pdf]

Glucose and NAADP trigger elementary intracellular β-cell Ca^{2+} signals

Paula Maria Heister[1,2], Trevor Powell[1] & Antony Galione[1]

[1] Department of Pharmacology, Mansfield Road, Oxford OX1 3QT, UK
[2] Department of Pathology, Addenbrooke's Hospital, Hills Road, Cambridge CB2 0QQ, UK

Supplementary Files

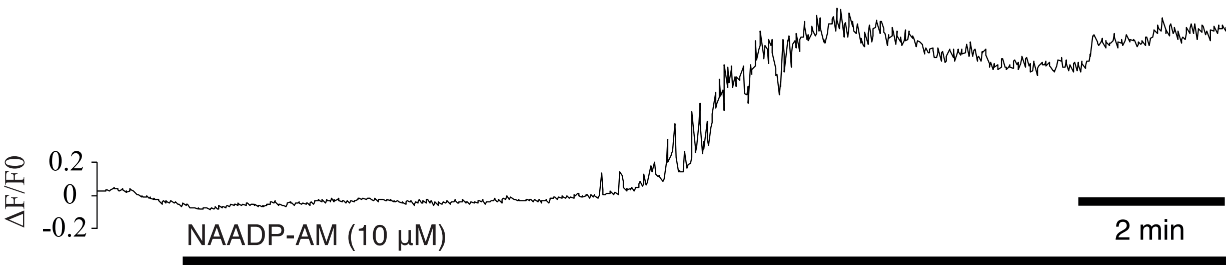

Figure S1 (Extended Data) β-cell sub-membrane Ca^{2+} response to NAADP-AM as recorded with TIRF. Representative trace of a submembrane calcium response to stimulation with 10µM NAADP-AM in the presence of 3mM glucose (used as baseline glucose in all experiments, see methods).

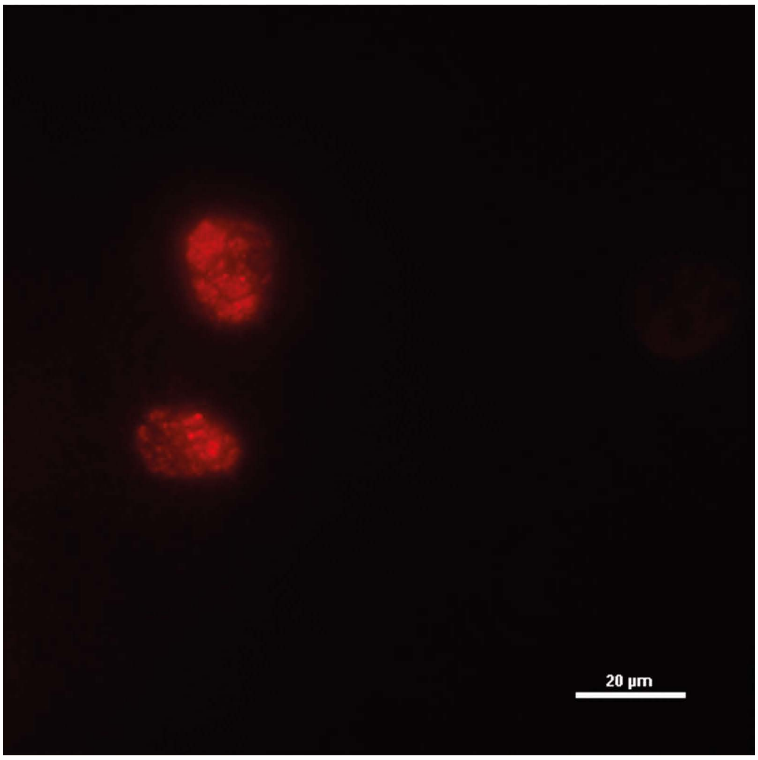

Figure S2 (Extended Data) Visualisation of acidic stores in the TIRF plane. LysoTracker Red labelling of β-cells as viewed withTIRF under 60 x magnification.

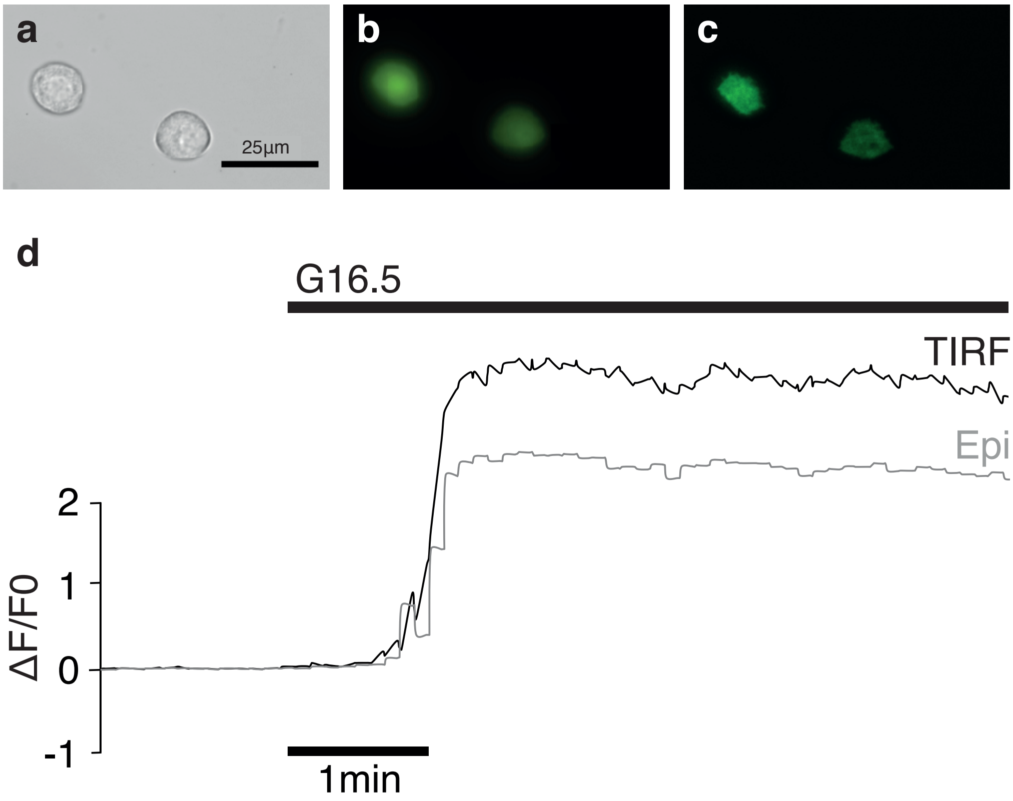

Figure S3 (Extended Data) Parallel imaging of TIRF and epifluorescence in β-cells. Examples of representative (a) brightfield, (b) epifluorescence, and (c) TIRF images of individual β-cells loaded with fluo-4 at 60 x magnification. (d) Representative trace of a parallel recording of whole-cell (epifluorescence; grey trace) and submembrane (TIRF; black trace) Ca^{2+} in response to 16.5 mM glucose in a β-cell.

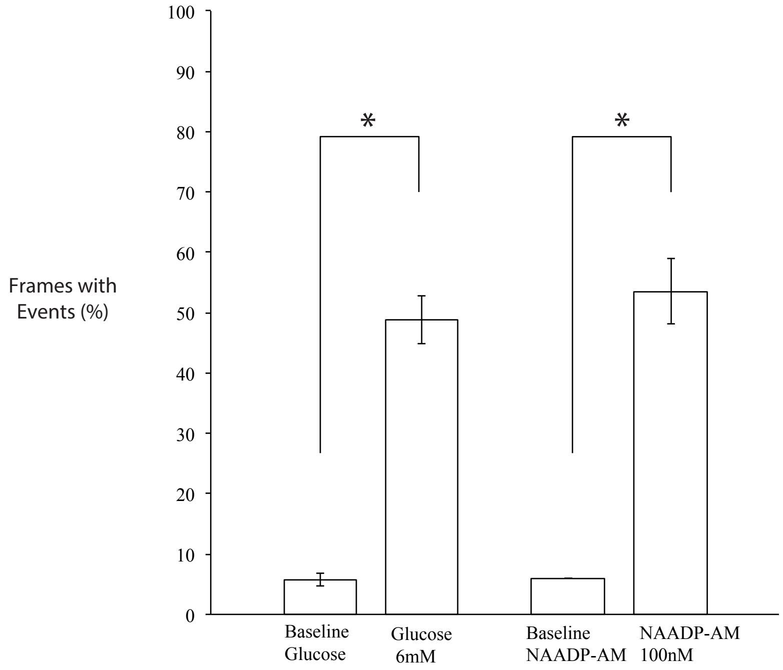

Figure S4 (Extended Data) Quantification of calcium release events in the presence of high EGTA (5mM). Percentage of frames showing events (defined as events of an amplitude more than 2 standard deviations above the baseline mean) before (baseline) and after stimulation with glucose (6mM) or NAADP-AM (100 nM) in the presence of 5mM EGTA. Results are from 4 cells (4 experiments, 2 animals) and 2 cells (2 experiments, 2 animals) respectively. * denotes significance (paired samples, one-tailed Student's t-test, $p<0.01$, $p<0.05$, respectively).

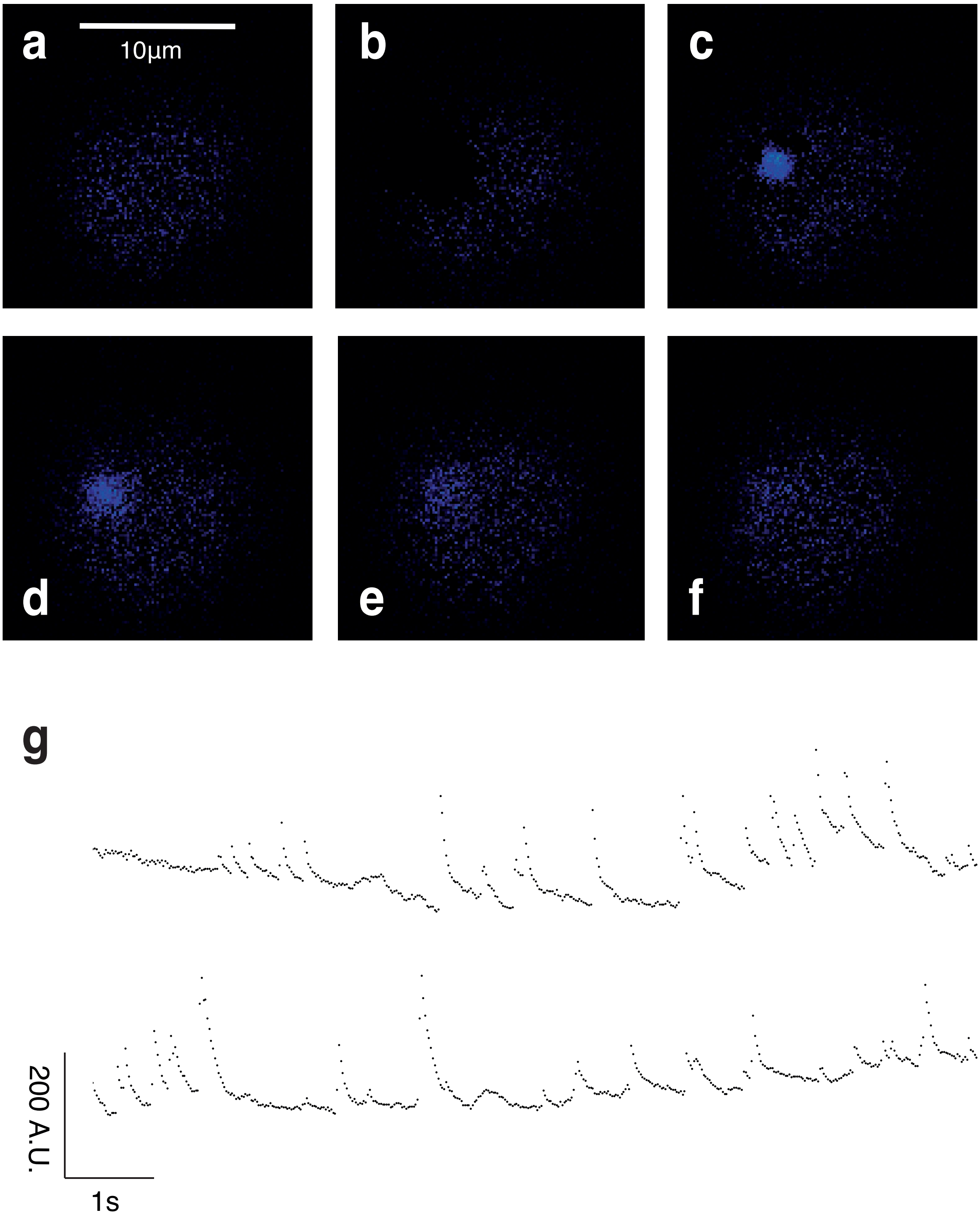

Figure S5 (Extended Data) High speed recordings of calcium events for illustration of diameter and time course. Cells were investigated as above but recorded at ~ 46 frames/second (hardware limit for the setup; proof of method experiment, n = 2, 1 experiment, 1 animal). (a – f) Images of a β-cell over the time course of one individual localised calcium event. (g) fluorescence intensity over time for two individual β-cells (note: traces not normalised, y-axis in arbitrary units, A. U.)

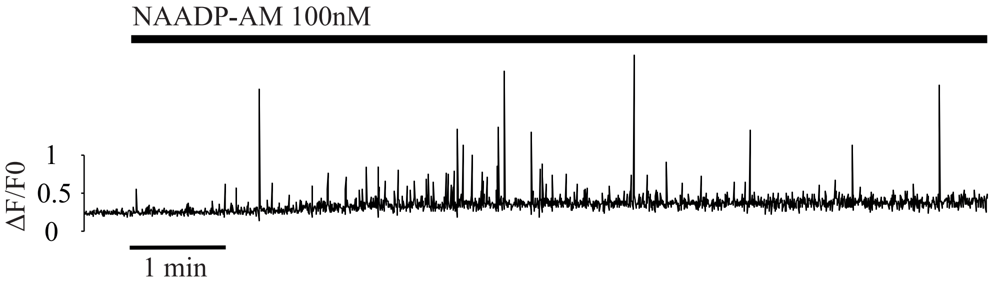

Figure S6 (Extended Data) Calcium release events increase and then decrease after stimulation. Representative TIRF trace of a ß-cell stimulated with 100 nM NAADP-AM in the presence of low EGTA after preincubation with thapsigargin. Maximum intensity change of subsequent frames after normalising to baseline plotted against time.

Supplementary Movie Legends

Movie 1 (Extended Data) First recording of elementary events in a pancreatic β-cell cluster in response to NAADP-AM (100 nM) visualised using TIRF microscopy at 100 x magnification. At the end of the experiment, extracellular Ca^{2+} is re-admitted, demonstrating a global Ca^{2+} response and cell viability. Recording speed: 3.3Hz, playback speed increased for ease of viewing.

Movie 2 (Extended Data) Recording of elementary calcium events in two pancreatic β-cells recorded at RAM capture speed (~ 46Hz) following spark detection (proof of method experiment, n = 2, 1 experiment, 1 animal).
